# Analytical Tools for the Study of Cellular Glycosylation in the Immune System

**DOI:** 10.3389/fimmu.2013.00451

**Published:** 2013-12-11

**Authors:** Yvette van Kooyk, Hakan Kalay, Juan J. Garcia-Vallejo

**Affiliations:** ^1^Department of Molecular Cell Biology and Immunology, VU University Medical Center, Amsterdam, Netherlands

**Keywords:** glycan analysis, glycosyltransferases, glycans, lectins, immune cells

## Abstract

It is becoming increasingly clear that glycosylation plays important role in intercellular communication within the immune system. Glycosylation-dependent interactions are crucial for the innate and adaptive immune system and regulate immune cell trafficking, synapse formation, activation, and survival. These functions take place by the *cis* or *trans* interaction of lectins with glycans. Classical immunological and biochemical methods have been used for the study of lectin function; however, the investigation of their counterparts, glycans, requires very specialized methodologies that have been extensively developed in the past decade within the Glycobiology scientific community. This mini-review intends to summarize the available technology for the study of glycan biosynthesis, its regulation and characterization for their application to the study of glycans in immunology.

## Introduction

Glycosylation is the most common post-translational modification of proteins. It is often estimated that more than 50% of all mammalian cellular and membrane-bound proteins are glycosylated, implicating an essential role in protein and cell function for carbohydrates. Indeed, carbohydrates play multiple roles in glycoprotein function: they participate in folding and maturation, contribute to the structural properties of glycoproteins, provide charge and hydrophilicity, and mediate interactions. In particular, carbohydrate-mediated interactions are specially crucial for the immune system (1). Glycans have been involved in the generation and loading of antigenic peptides into MHC-I (2), immune cell trafficking (3), T cell receptor signaling and apoptosis (4), B-cell receptor signaling (5), antibody function (6), immune cell differentiation (7), pathogen recognition (8), and immune homeostasis (9). Therefore, determining glycan structure, their biosynthetic regulation, their aglycon, and their binding partners is a fundamental step toward understanding the role of glycosylation in the immune system.

**Figure 1:**
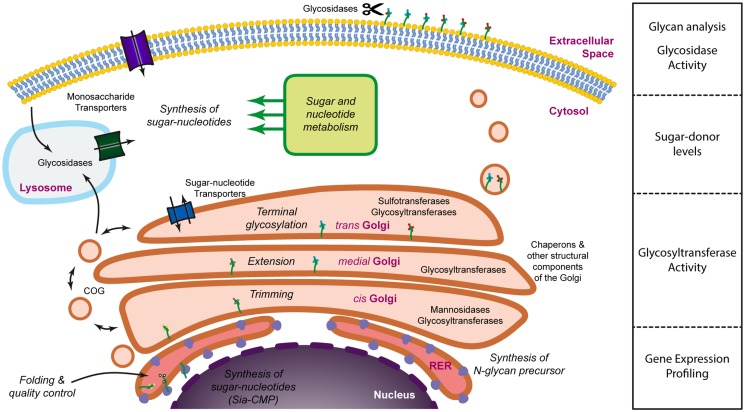
**Dissecting the glycosylation machinery**. Glycosylation is a complex process that involves a large number of molecules and organelles. The glycosylation machinery can be defined as the set of enzymes, chaperones, transporters, sugar donors, and accessory molecules necessary for the modification of proteins or lipids with carbohydrates. Since many of these molecules are subjected to regulation, glycosylation is a highly dynamic process and it is, therefore, interesting to address not only the array of glycans present on the cell surface or the secretome, but also the activity and the expression levels of the molecules involved in glycan biosynthesis.

Glycans are often defined as assemblies of carbohydrates that include monosaccharides, oligosaccharides, polysaccharides, and their conjugates (glycoproteins, glycolipids, and proteoglycans). The structural diversity of glycans depends on several factors, namely differences in monosaccharide composition, anomeric state, glycosidic linkage, branching, the presence of non-carbohydrate substituted components (phosphorylation, sulfation, acetylation, etc.) and linkage to their aglycones (peptide, lipid, etc.) (10). Each of these structural factors is ultimately determined during glycan biosynthesis by the relative composition of the glycosylation machinery. The term “*glycosylation machinery*” refers to the set of, mainly enzymes, but also co-factors, transporters, and activated sugar donors that are necessary for the natural biosynthesis of glycans. It has been estimated that approximately 1% of the genome is dedicated to glycosyltransferases (11) and, if all genes involved in the glycosylation machinery are considered, this figure would probably rise to approximately 3–4%, thus a significant proportion. The glycosylation machinery is not localized to a single specific organelle within the cell and should be envisioned as a virtual engine (Figure 1) which involves mainly the Golgi apparatus, but also several other organelles and intracellular compartments, such as the nucleus (sialic acid biosynthesis), the endoplasmic reticulum (initial steps of *N*-glycosylation), lysosomes (monosaccharide recycling), or the cytoplasm (sugar donor and *N*-glycan precursor biosynthesis). With such a widespread localization and the involvement of so many factors it is no surprise that several levels of regulation have been described that affect the glycosylation process. Central to the glycosylation process, many glycosyltransferases have been shown to be regulated through transcriptional (12), post-transcriptional (13, 14), and post-translational (15) mechanisms. In addition, the activity of some glycosyltransferases may also be regulated through the interaction with chaperons (16, 17), competition for substrate with other glycosyltransferases (18), the availability of sugar donors (19), the pH at the Golgi (20), cleavage of their transmembrane domain (21), or even relocation to different organelles (22). Also, the regulation of the expression of glycoproteins as well as their modification by glycosidases (23) once on the cell membrane or the extracellular space contribute to the regulation of glycosylation. These mechanisms may operate in response to physiological (24–26) or pathological (27–29) cues and often have a biological correlate that is dependent on changes in the interaction with glycan-binding proteins (30). Thus, glycosylation is a highly regulated process that is extremely sensitive to both intracellular and extracellular stimuli. Moreover, due to the nature of the glycosylation process, the resulting glycoproteins exist as a mix of the same peptide backbone with a variety of different glycans. The diversity of these glycans depends on the relative composition of the glycosyltransferases expressed and the interplay of all the regulatory stimuli that operate at a particular moment. This can affect both the number of glycans attached per glycoprotein, a type of variation that is referred to as macroheterogeneity, as well as the nature of these glycan chains (known as microheterogeneity). Thus, glycoproteins usually exist as complex mixtures of glycosylated variants or glycoforms. As an example, the human erythrocyte molecule CD59 consists of more than 120 different glycoforms, despite having a single *N*-linked glycosylation site and a couple of potential *O*-linked glycosylation sites (31).

Unfortunately, we still lack a systems biology approach to allow the modeling of the glycosylation machinery. Such a model would be extremely useful to predict how changes in the relative expression of different components of the glycosylation machinery would lead to alterations in the glycan profile of cells or secreted proteins. Accumulating evidence demonstrates, nevertheless, that there is a good correlation between changes in the transcript levels of glycosyltransferases and differences in the glycosylation pattern, suggesting that the modeling of the glycosylation machinery could be a possibility in the future. Until then, a comprehensive analysis of cellular glycosylation should incorporate different types of methodologies that provide information on the expression of the different components of the glycosylation machinery, their activity, as well as the characterization of the secreted or membrane-bound glycome (Figure 1).

Considering the different regulatory checkpoints of the glycosylation machinery, the most logical and accessible assays to address the glycosylation of cells would be the gene/protein expression profile of key components of the glycosylation machinery, their activity, and the glycosylation profile. We will now discuss the different methodological approaches to each of these types of assays, especially in the context of the study of the glycosylation of immune cells.

## Gene-Expression Analysis

The majority of the human and mouse glycosyltransferases known to date were cloned and characterized between the late 80s and the early decade of this century. The development of gene-expression technologies such as microarray technology and real-time PCR coincided with the completion of the list of existing glycosyltransferases and it is, therefore, no surprise that efforts were made to specifically develop gene-expression microarray-based methods to adequately address the glycosylation-related transcriptome. One of the most extensively used microarrays has been the glycogene-chip developed by the Consortium for Functional Glycomics. The last version of this microarray contained probes for more than 1200 human and mouse glycosylation-related genes, including glycosyltransferases (256), glycan-binding proteins (146), glycosidases (88), nucleotide-sugar synthesizing enzymes and transporters (77), and conserved oligomeric Golgi (COG) complex proteins. In addition, several immune-related molecules such as interleukins, chemokines, and growth factors with their respective receptors were included, making this microarray extremely interesting for the analysis of the transcriptome of different immune subpopulations. In order to enhance specificity, this microarray consisted of 25 probes per gene. Unfortunately, due to the conclusion of the 10-year Glue Grant from the National Institute of General Medical Sciences (NIGMS), production of this microarray has been discontinued, although the data remains publicly available at the website of the Consortium for Functional Glycomics (http://www.functionalglycomics.org/glycomics/publicdata/microarray.jsp). Alternatives to the use of this microarray are genome-wide microarrays (Illumina microarrays also provide quantification based on 20–30 probes per gene) and real-time PCR of selected genes. Some currently available microarray platforms, like Illumina, provide genome-wide microarrays with also a high number of probes per gene. Analysis of the expression of genes encoding for glycosylation-related enzymes on data generated using this type of microarrays should be able to provide information to predict what type of glycans are to be expected on the cell of interest or what kind of glycosylation changes may operate under the treatment of study. In addition, since the whole genome is covered, these microarrays may be helpful in addressing the molecular mechanisms responsible for the regulation of the glycosylation-related gene-expression changes. Still, the use of low-density screening methods, such as real-time PCR (32–34) can be quite informative depending on the research question. The advent of next-generation sequencing technologies (35) will surely provide additional possibilities for quantification of glycosylation-related gene expression, with the advantage to identify mutations/splice variants and epigenetic variation associated with the glycosylation-related genes, potentially leading to the identification of susceptibility markers and inherited disease traits, a concept that has previously been suggested for autoimmunity (36, 37).

## Glycosyltransferase and Glycosidase Activity Assays

As already mentioned, glycosyltransferases may be regulated at the expression level, but also, since they are enzymes, in their catalytic activity. Several factors may contribute to this, including pH, substrate availability, interaction with co-factors or chaperons, and post-translational modifications affecting activity. Thus, determining the activity of glycosyltransferases and glycosidases *in vitro* provides a new layer of information to the study of their regulation and also facilitates the identification of specific inhibitors. However, glycosyltransferase assays (38, 39) are complicated by the fact that all Leloir-type glycosyltransferases (sugar-nucleotide dependent glycosyltransferases) that transfer the same sugar use the same sugar-nucleotide donor, but can differ in their acceptor specificity, and in the regio- and stereochemistry of the transfer reaction. In addition, glycosyltransferases can be rather promiscuous in their acceptor specificity (40). In general, the activity of glycosyltransferases can be monitored by following either the depletion of the sugar donor and the substrate(s) or the formation of the reaction products, whereas glycosidase activity is detected by following the loss of substrate. In order to allow the monitoring many assays make use of radiochemically- or fluorescently-tagged donor or acceptor analogs. Then, chromatographic, radiochemical, spectrophotometric, or immunological techniques are used to separate and/or detect one or more of the reaction species. Although glycosyltransferase activity assays have helped enormously in the characterization of glycosyltransferases and the identification of glycosyltransferase inhibitors, their contribution to understanding the regulation of glycosylation is limited. This limitation depends on the fact that many of the glycosyltransferase assays are based in reagents that are not able to cross membranes and, therefore, cannot be used in living cells or organisms. Alternatively, metabolic labeling approaches have been developed that allow the tagging of newly synthesized glycoproteins with radiochemically labeled glycans. Most recently, the use of bioorthogonal chemical reporters has allowed metabolic glycan labeling even *in vivo* (41). Importantly, the reporter must be non-toxic and small enough to not interfere with the transport of the monosaccharide into the cell, its incorporation into a sugar donor and the glycosyltransferase reaction. This is the case of azido or alkynyl monosaccharide derivatives, which have been used for the labeling of most glycan subtypes, except for glycosaminoglycans and glycosylphosphatidylinositol anchors (41). Unfortunately, monitoring of specific glycosyltransferases is not possible using this technology, but it can still be very useful to address the effect of multiple biological stimuli on specific glycan subtypes (e.g., sialylation, fucosylation, *O*-glycans, etc).

## Glycan Analysis

The complete characterization of the glycans from cell membranes or purified glycoproteins is a process that involves dedicated Analytical Chemistry technology and requires the integration of different analytical approaches. However, it is not always necessary to perform a comprehensive glycan sequencing and, depending on the type of experimental set up and evidence required, fast and simple approaches such as lectin binding assays may be sufficient. The availability of a large set of plant lectins with defined specificity has allowed the development of simple assays for the high-throughput gross characterization of the glycosylation of cells or purified glycoproteins (42). Small scale screening using selected lectins can easily be set up as flow cytometry or ELISA assays. On the other hand, lectin microarrays are becoming increasingly popular, specially in the development of disease-related biomarkers in cancer (43, 44). Unfortunately, most lectins have basic preferences to a broad set of carbohydrate structures or epitopes and a certain level of cross-reactivity is often observed. Therefore, lectins are not very practical when a detailed glycan characterization is needed. In this case, glycans can be sequenced by several different but complementary approaches. The most extended methodology is based in the purification of glycans after chemical or enzymatic released from their aglycon. This is considerably easier for *N*-linked glycans, which can be enzymatically released from mammalian glycoproteins using an amidase (PNGase F) (45). Unfortunately, only one enzyme has been described so far to be able to cleave the core 1 *O*-glycan, endo-α-*N*-acetylgalactosaminidase (*O*-glycanase), but not its extended variants or any of the seven remaining *O*-glycan core structures (46, 47). Alternatively, chemical methods such as hydrazinolysis (48), deglycosylation by anhydrous trifluoromethanesulphonic acid (49), or non-reductive alkaline β-elimination (50) can be used instead, although these reactions require careful optimization to prevent glycan degradation (51). Regardless of the method used, released glycans can then be purified and analyzed by chromatographic and/or mass spectrometric methods. Small glycans can directly be analyzed by means of high performance anion-exchange chromatography with pulsed amperometric detection in stand-alone mode (52) or online-coupled to mass spectrometry through a desalter unit (53). High-performance anion-exchange chromatography with pulsed amperometric detection can also be used for monosaccharide analysis of purified glycans (54), which can be useful as an aid for further characterization, but requires high concentrations of experimental sample. Most often, glycans purified after deglycosylation are derivatized at their reducing end with a fluorochrome (55) and then resolved by hydrophilic interaction chromatography with a fluorescence detector. Further characterization is achieved by sequential deglycosylation using exoglycosidases (56), which specifically cleave glycosidic bonds of individual monosaccharide units from the terminal residue. Exoglycosidase digestion results in a shift in the glucose-unit value allowing detailed structural assignments with linkage information (56). Robotic systems and ultra-performance liquid chromatography in combination with sub 2 μm stationary phase capillary columns is allowing the implementation of very promising high-throughput glycan analysis projects that will certainly have an important impact in biomarker discovery (57, 58). In addition, the incorporation of an online mass spectrometer after the fluorescence detector facilitates glycan characterization without the need of extensive exoglycosidase reactions (55, 59). Alternatively, glycans can also be analyzed by porous graphitized carbon LC-MS/MS (60–63).

Derivatization of glycans with 9-aminopyrene-1,3,6-trisulfonic acid (APTS) or 8-aminonaphthalene-1,3,6-trisulfonic acid (ANTS) provides glycans with electrophoretic mobility and fluorescence detection, allowing their separation by capillary electrophoresis coupled to a laser-induced fluorescence detector (64). The main advantages of this technology are its sensitivity (10^−15^ to 10^−18^ mol of oligosaccharide samples), short separation time (<20 min), and high-throughput potential and, when combined with mass spectrometry, this method provides simultaneous glycan characterization (65).

Glycans can also be directly analyzed by mass spectrometry, with the advantage of providing a link between mass and composition. In order to perform mass spectrometric analysis of glycans it is necessary to derivatize them, since the ionization efficiency of glycans (especially those carrying terminal sialic acids) is generally low. Typical derivatization methods include permethylation (66), methyl-esterification of sialic acids (67), or the above-described fluorescent tagging of the reducing end. Often, rapid profiling is achieved through matrix-assisted laser desorption ionization time-of-flight mass spectrometry because it is fast, simple, and requires only a small amount of sample. Ion fragmentation through electrospray ionization mass spectrometry, collision-induced dissociation and MS/MS help in achieving structural characterization. More recently developed fragmenting technologies such as electron capture dissociation and electron transfer dissociation have created huge expectative for the implementation of top-down proteomics (68) and their application to glycomics and glycoproteomics. Approaches based on this technology would be ideal for the sequencing of *N*- and *O*-linked glycans together with their peptide assignment. Intact *N*- and *O*-glycopeptides from purified glycoproteins have already been successfully analyzed using this approach (61, 69–73), but methods for more complex samples such as cell lysates remain to be implemented. Importantly, the development of analysis software and glycan databases for the direct assignment of glycan structures to specific masses in different platforms is pushing the field forward by facilitating reporting and data mining (74–76).

## Concluding Remarks

Although the glycome of several immune cell populations has already been profiled (25, 26, 77) and accumulating evidence highlights the importance glycosylation regulation in multiple aspects of immune biology (78–81) we still need a better understanding of how glycosylation is regulated in different immune cell subpopulations. A better integration of glycobiological methodology in the immunological community is a pre-requisite, for which we hope this primer will be a useful first step.

## Conflict of Interest Statement

The authors declare that the research was conducted in the absence of any commercial or financial relationships that could be construed as a potential conflict of interest.
